# An Accessible Integrated Nanoparticle in a Metallic Hole Structure for Efficient Plasmonic Applications

**DOI:** 10.3390/ma15030792

**Published:** 2022-01-21

**Authors:** Vasanthan Devaraj, Jong-Wan Choi, Jong-Min Lee, Jin-Woo Oh

**Affiliations:** 1 Bio-IT Fusion Technology Research Institute, Pusan National University, Busan 46241, Korea; devarajvasanthan@gmail.com; 2 Department of Chemistry and Life Science, Sahmyook University, Seoul 01795, Korea; jchoi@syu.ac.kr; 3 School of Nanoconvergence Technology, Hallym University, Chuncheon 24252, Korea; 4 Center of Nano Convergence Technology, Hallym University, Chuncheon 24252, Korea; 5 Department of Nano Fusion Technology and BK21 Plus Nano Convergence Division, Pusan National University, Busan 46241, Korea; 6 Department of Nanoenergy Engineering, Pusan National University, Busan 46241, Korea

**Keywords:** simulations, optics, plasmonics, gap-mode enhancement, integrated nanostructure

## Abstract

Addressing the severe deterioration of gap mode properties in spherical-shaped nanoparticles (NPs) becomes necessary due to their utilization in a wide range of multi-disciplinary applications. In this work, we report an integrated plasmonic nanostructure based on a spherical-shaped nanoparticle (NP) in a metallic hole as an alternative to a NP-only structure. With the help of three-dimensional (3D) electromagnetic simulations, we reveal that when a NP is positioned on the top of a metallic hole, it can exhibit superior gap-mode-based local-field intensity enhancement. The integrated nanostructure displayed a ~22-times increase in near-field enhancement characteristics, similar to cube- or disk-shaped nanostructure’s plasmonic properties. From an experimental perspective, the NP positioning on top of the metallic hole can be realized more easily, facilitating a simple fabrication meriting our design approach. In addition to the above advantages, a good geometrical tolerance (metallic hole-gap size error of ~20 nm) supported by gap mode characteristics enhances flexibility in fabrication. These combined advantages from an integrated plasmonic nanostructure can resolve spherical-shaped NP disadvantages as an individual nanostructure and enhance its utilization in multi-disciplinary applications.

## 1. Introduction

A methodology to create and design nanostructures or devices before fabrication will save time and cost in developing efficient applications in various fields [1,2,3,4,5]. One such field that merited from this modeling approach is optics, where attractive applications in plasmonics, photonics, non-classical light sources, quantum dots, semiconductors, and so on can be realized [4,6,7,8,9,10]. Notably from those applications, plasmonic nanostructures were studied extensively, which reveals unique optical properties such as the negative index of refraction, complex sub-wavelength characteristics, extraordinary transmission, tunable resonances, photocatalytic activity, and color printing [10,11,12,13,14,15]. In particular, exploiting interactions between light and matter with the help of surface plasmon resonance (SPR) results in variety of properties and functions. This SPR can be categorized into two parts: surface plasmon polariton (SPP)—the propagation of electron oscillations along the planar interface; and localized SPR (LSPR)—the confinement of electron oscillations on a subwavelength structure. These two forms of SPR, once excited, can lead to remarkable enhancement of local field or hot-spots and allows manipulation of light below the diffraction limit. This remarkable property of SPR helped in yielding a diverse range of applications such as artificial magnetism, imaging, sensing, energy, optical switching, and photodetection [1,4,6,16,17,18,19,20,21].

Numerical modeling approaches based on three-dimensional electromagnetic simulations created a positive impact in achieving better plasmonic devices by exploiting a variety of geometrical designs [1,4,6,10,14,15,16]. Of many features, the following will be key goals of the modeling approach: to develop efficient plasmonic nanostructures; the availability of geometrical tolerance with minimal loss in plasmonic properties; and non-complex low-cost fabrication [1,8,9,15]. Metallic nanoparticles (NPs) are one such example where there is a possibility of realizing attractive plasmonic characteristics through a simplistic approach [4,10,22,23,24,25,26]. In particular, spherically shaped NPs have been studied extensively (experiment and theory) owing to their simplicity in fabrication, and ease of use in versatile applications. Despite the advantages mentioned above, rapidly deteriorating plasmonic characteristics from spherical NPs were observed on the presence of minor nanoscale geometrical errors [27,28,29,30]. Due to these issues, alternative geometries with respect to sphere-like disk and cube were preferred due to their excellent optical nature. Even though a NP-on-mirror (NPOM) design can enhance spherical NPs’ optical properties, gap plasmonic properties become absent even with gap sizes exceeding ≥6 nm [27]. It is necessary to address this issue, where a simple and effective nanostructure involving spherical NPs can be realized. With the aid of numerical modeling, it is possible to envision efficient spherical NP plasmonic nanostructures facilitating simple fabrication [10,15,18,19,20].

In this work, we report an integrated nanostructure (NP in a metallic hole on a SiO_2_ substrate) which can be realized practically. The primary purpose of this simulation study is to reveal its plasmonic characteristics and to discuss its optical properties and possible advantages by utilizing this integrated nanostructure. We believe our work can act as a design platform, exploiting meaningful insights and thereby coalescing this integrated structure for multi-disciplinary applications.

## 2. Materials and Methods

**Figure 1:**
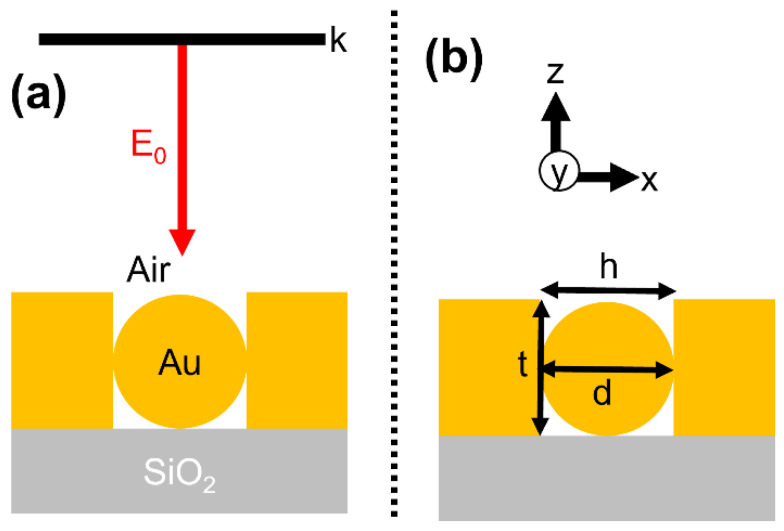
(**a**) Cross-sectional schematic of nanoparticle in a hole structure on a substrate describing simulation and material properties. A broadband plane-wave source with an incident electric field of E_0_ is used to illuminate the nanostructure to understand its plasmonic properties. (**b**) Description of the nanostructure’s geometrical parameters.

Optical modeling is carried out using the three-dimensional (3D) finite-difference time-domain (FDTD) method (Lumerical FDTD solutions, ANSYS Inc. Vancouver, Canada). The modeled structure consists of a metallic hole with a nanoparticle (NP) on a SiO_2_ substrate (Figure 1a). To begin with, we simplified the model consisting of a single hole with a single NP. For both the hole and NP, we used gold as a material. The geometrical parameters are as follows: hole’s width and thickness are given by “h” and “t,” respectively; NP diameter is given by “d” (Figure 1b). For most of this study, we used the following conditions: d = 100 nm; h = 100 nm; and t = 100 nm. In the case of geometrical tolerance analysis, the condition is set to h ≠ t. The 3D structure is surrounded by perfectly matched layer (PML) boundary conditions in XYZ directions. A broadband plane-wave source is used to excite the structure in a normal direction from the top (+Z) with an incident field of “E_0_” and wave vector “k”. Meshing conditions: “5 nm mesh covering the total simulation area and a 0.3 nm mesh-override size surrounding nanostructure of our main concern”. The following refractive indices are used: Johnson and Christy database for gold and Palik database for SiO_2_ [31,32]. Air surrounds the nanostructure on top with a refractive index of *n* = 1.

A box-shaped power monitor is placed close to the nanostructure to record the near-field enhancement. Approximately, the fourth power of near field or |E/E_0_|^4^ can be compared to the electromagnetic enhancement factor of SERS (for clarity in linear scale graphing, we used |E/E_0_|). By assuming the Raman probe molecules are distributed randomly and uniformly on the NP surface, an averaged electromagnetic enhancement factor can be calculated by averaging the volume integral of |E/E_0_| [1,4,6,8,10,27,29,30,33,34,35,36,37,38,39]:

(1) $$\mathrm{Maximum}\;\mathrm{near}-\mathrm{field}\;\mathrm{enhancement}=\frac{\int \int \int \left|E/E_{0}\right|\mathrm{dV}}{V}\;$$

As seen from Equation (1), E_0_ is the modulus of the incident electric field (|E_0_| = 1 V/m), E represents the generated local field (E = E_x_, E_y_, E_z_), and V is the volume at a certain distance (here we considered 2 nm) above the metallic surface [1,29,40,41].

To identify and interpret complex plasmonic modes, we utilized three-dimensional surface charge density mappings (3DSCDM) using COMSOL Multiphysics software (Wave optics module). By considering skin effect, an integration of Gauss’s law, outward normal vector, and local electric field, 3D surface charge density “ρ” is calculated [1,29,40,41].

(2) $$\rho =\frac{\varepsilon_{0}\;\left(n_{x}\;\cdot \;E_{x}+n_{y}\;\cdot \;E_{y}+n_{z}\;\cdot \;E_{z}\right)}{\delta \left(1-e^{-R/\delta }\right)}\;\alpha \;\left(n_{x}\;\cdot \;E_{x}+n_{y}\;\cdot \;E_{y}+n_{z}\;\cdot \;E_{z}\right)$$

From Equation (2), radius of NP is given as “*R*”, outward normal vector “*n*”, skin depth “*δ*” and permittivity of vacuum “*ε*_0_”.

## 3. Results and Discussion

### 3.1. Problems with Individual Nanostructure(s)

**Figure 2:**
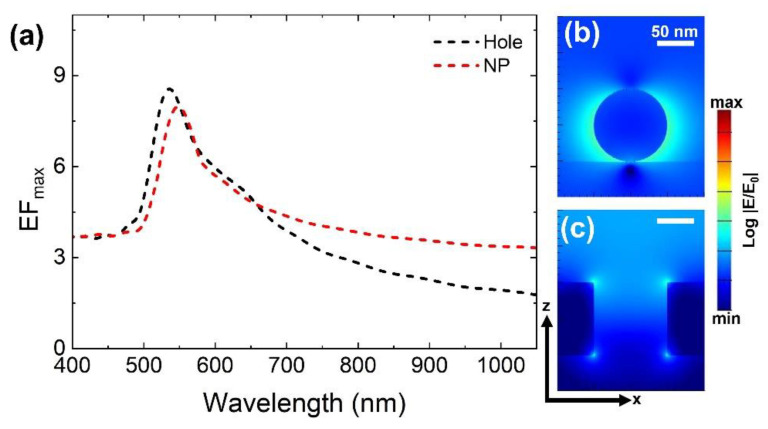
(**a**) Near-field enhancement EF_max_ spectral properties taken from individual NP and metallic hole on SiO_2_ substrate. Extracted cross-sectional XZ electric field profiles from their resonance wavelength position of NP structure (**b**) and metallic hole structure (**c**) at 550 nm and 535 nm, respectively. Scale bars in Figure 2b,c correspond to 50 nm.

Figure 2 displays the fundamental issue concerning individual nanostructures either in the form of NP or a metallic hole on a glass or SiO_2_ substrate. We did not use a metallic mirror as a substrate, to clearly evaluate and distinguish the individual nanostructure’s problems. Broadband near-field spectra reveals a EF_max_ of ~8 can be extracted from individual nanostructures (Figure 2a). Cross-sectional electric field profiles reveal no trace of gap mode characteristics from these individual nanostructures (Figure 2b,c) extracted at 550 nm and 535 nm wavelength positions for NP and hole structures, respectively. NP mode or hole modes are solely identified from these individual nanostructures on a SiO_2_ substrate (unless there is a possibility of an increased number of NPs or with extremely smaller metallic hole width “h” size). In case of NPs, even in the presence of a metallic mirror substrate (NPOM design), gap mode deteriorates rapidly and diminishes when spacer thickness becomes ≥6 nm [27]. Presenting a plasmonic nanostructure design with good geometrical tolerance facilitating flexible fabrication and enhanced gap mode properties will be necessary in developing efficient applications.

### 3.2. Integrated Nanostructure: Nanoparticle in a Metallic Hole

**Figure 3:**
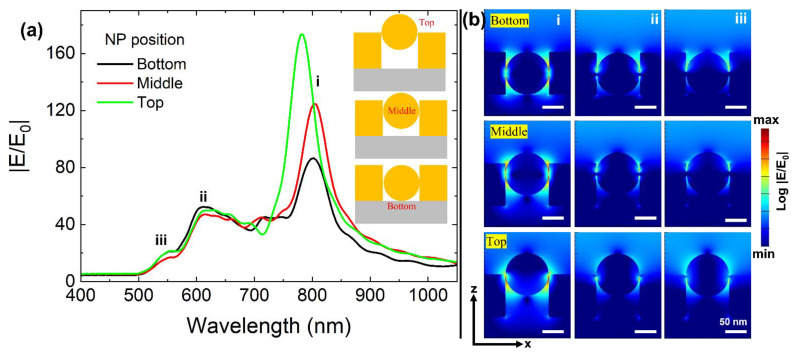
(**a**) Maximum near-field enhancement EF_max_ properties for three different NP positions in a hole structure (top, middle, and bottom) and corresponding cross-sectional XZ electric field profiles (**b**) extracted from positions i, ii, and iii. All scale bars in Figure 2b corresponds to 50 nm.

Here, we introduce an integrated plasmonic nanostructure (NP in a metallic hole) on a SiO_2_ substrate as an alternative geometrical design to enhance plasmonic properties. In the first part of the optical analysis, we evaluated how the position of the NP in the hole determines the plasmonic properties. Following geometrical conditions used in this section: h = t = d. We applied these geometrical conditions for the following requirements: position of NP on the top of the hole (suitable with a state of h = d); simplification of a model (h = t = d); and flexible understanding of plasmonic properties from our structure. The inset Figure 3a displays the three modeled NP positions: bottom, middle, and top. In practical realization, a NP positioned on the top will be easier to fabricate but, at the same time, there can be a minor probability of the NP’s movement towards the bottom. By considering the possibilities of NP locations, we compared top, middle, and bottom conditions. Figure 3a displays the near field enhancement EF_max_ spectra from these nanostructures. We marked three dominant resonance peak positions from “i” to “iii” to interpret the origin of plasmonic modes. Superior EF_max_ properties are revealed when NP is positioned at the top of the metallic hole. The bottom position displays comparatively lower EF_max_ values from its resonance wavelength marked as “i”. The near field EF_max_ properties deteriorated from 173, 125, and 87 when the NP position moved from top to the bottom. The wavelength position marked at “i” is chosen to distinguish primary plasmonic properties due to its dominant gap mode characteristics, which are revealed from the cross-sectional XZ electric field profiles (Figure 3b). Highly concentrated, enhanced near-field spots can be seen at metal–metal contact positions (with the better case being the NP positioned at top) in the case of “i,” explaining its dominant gap mode origin when compared to “ii” and “iii”. In comparison to “i”, weakly enhanced near-field spots can be seen at metal–metal contact positions at “ii”, thereby assigning it to “supporting gap mode”. NP mode is observed at “iii” as there is no trace of enhanced local field at metal–metal contact [27,29].

**Figure 4:**
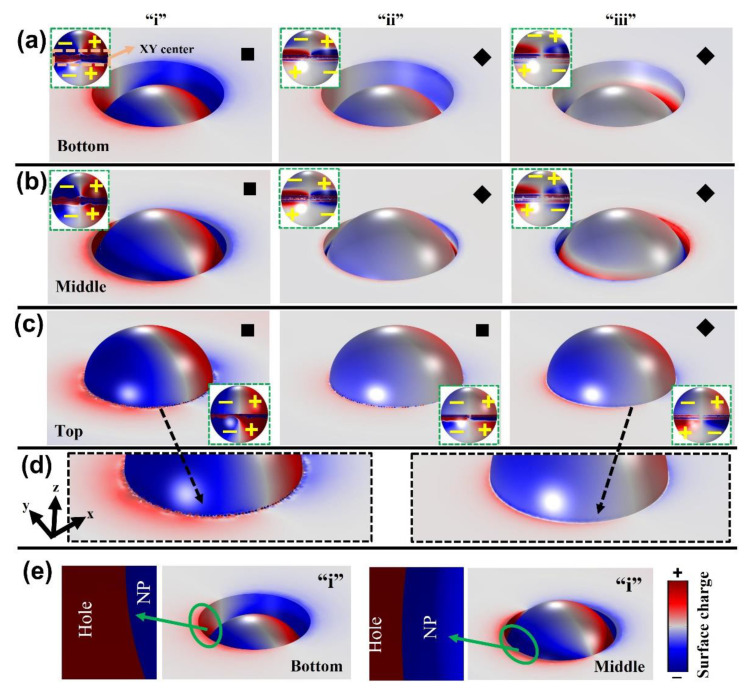
(**a**–**c**) 3DSCDM profiles extracted from integrated nanostructures from their respective resonance wavelength positions marked from “i” to “iii”. (**d**) Magnified surface charge density distributions near NP–hole interface for an integrated nanostructure with a NP positioned on the top (for resonance wavelength positions “i” and “iii”). (**e**) Magnified surface charge density distributions near NP-hole interface for integrated nanostructures when NP is positioned at bottom and middle (resonance wavelength position “i”).

It will be difficult to judge plasmonic properties solely based on cross-sectional electric field profiles. Utilizing 3DSCDM calculations will be helpful in understanding the optical properties in detail. 3DSCDM profiles for integrated nanostructures taken at resonance wavelength positions marked from “i” to “iii” are displayed in Figure 4. To explain plasmonic modes in an easier way, we used the following symbols: a solid square symbol for presence of the dipole mode and a solid diamond symbol for the quadrupole mode [1]. Figure 4a–c shows 3DSCDM profiles for integrated nanostructures with the NP positioned from bottom, middle to top, respectively (green dotted inset figure displays surface charge mappings in NP for the respective integrated nanostructures). For “i” wavelength position, dipole mode is clearly visible for all the three integrated nanostructures. Differences in plasmonic modes appear for “ii” and “iii” positions. For both bottom and middle NP positions, the quadrupole mode is noted as “ii” and “iii”. However, for the top NP position, it differed: the dipole mode appears at “ii” and quadrupole mode at “iii”. In addition, for the top NP position, it is significant to note that whispering gallery mode-like surface charge interactions (Figure 4c,d, observed at resonance wavelengths “i” and “ii”; absent for “iii”) could be seen at the NP–hole interface, a probable reason for higher EF_max_ contribution as compared to middle and bottom NP positions. No such whispering gallery mode-like surface charge interactions could be seen with the middle and bottom NP positions (Figure 4e); thus, we can conclude that when a strong coupling between a brighter (dipole) gap mode and whispering gallery mode-like surface charge interactions (at NP-hole interface) happens, it can generate a highly localized field from the integrated nanostructure.

Surface charge interactions at the center of NP (XY direction) in the integrated nanostructures happen because of NP–hole contact (orange dotted line from the inset Figure 4a). When closely observed, the NP center (in XY direction) is inside the hole for middle and bottom positions; hence, circular-line patterned surface charge interactions are presented on either side of XY center. But in the case of the top NP position, the NP (XY) center lies at hole interface; thus, the circular-line patterned surface charge interactions are presented at XY center and below only. Overall, two gap modes (brighter dipole mode) are observed for the top NP position as opposed to a single gap mode for middle and bottom NP integrated nanostructures (dark mode or quadrupole mode observed at “ii”). Justification of “iii” as NP mode can explained on basis of individual NP’s resonance and smaller EF_max_.

The assignment of gap modes for marked positions “i” and “ii” from integrated nanostructures in Figure 3a can be justified by comparing it to individual nanostructures (Figure 2) with the following characteristics: highly enhanced EF_max_ values; brighter local-field spots originating at metal–metal contacts; and red-shifted wavelength positions. Critically, at the same time, an integrated nanostructure with the top NP position displayed better EF_max_ properties based upon two brighter gap modes (dipole mode) in combination with whispering gallery mode-like charge properties at the NP–hole interface. Significantly, integrated nanostructures exhibited a maximum of a ~22-times increase in near-field enhancement EF_max_ when compared with individual nanostructure(s), which is critically advantageous to the nano-structural design.

**Figure 5:**
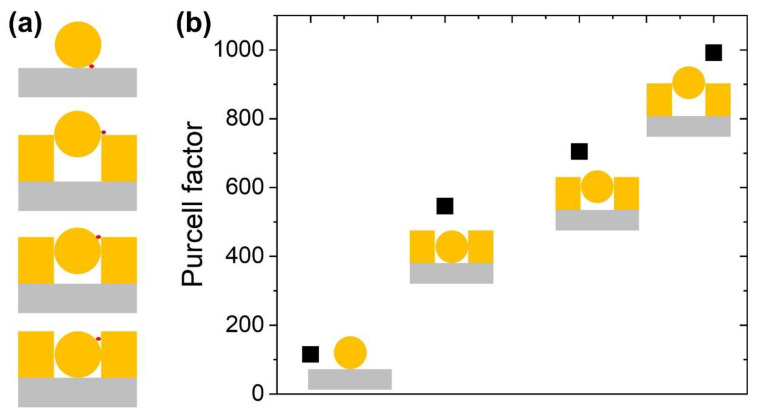
(**a**) Schematic position of an emitter (solid red color dot) placed close to “hot-spot” or EF_max_ region and extracted Purcell factor (**b**).

To further strengthen the importance of the integrated nanostructure, we calculated the Purcell factor in understanding the local intensity enhancement. We had extracted the Purcell factor from integrated nanostructures and compared it with individual NP structure (Figure 5). For this purpose, we had placed the emitter close to the NP’s EF_max_ area as schematically shown in Figure 5a [42,43]. The Purcell enhancement is ~10 times better with NP positioned at the top (integrated nanostructure) as compared to the individual nanostructure. Combination of higher Purcell factor and local field enhancement properties from the integrated nanostructure (with NP on the top) will benefit surface-enhanced applications, cavity nanostructures, sensing, and so on [44].

### 3.3. Geometrical Tolerance of Metallic Hole

**Figure 6:**
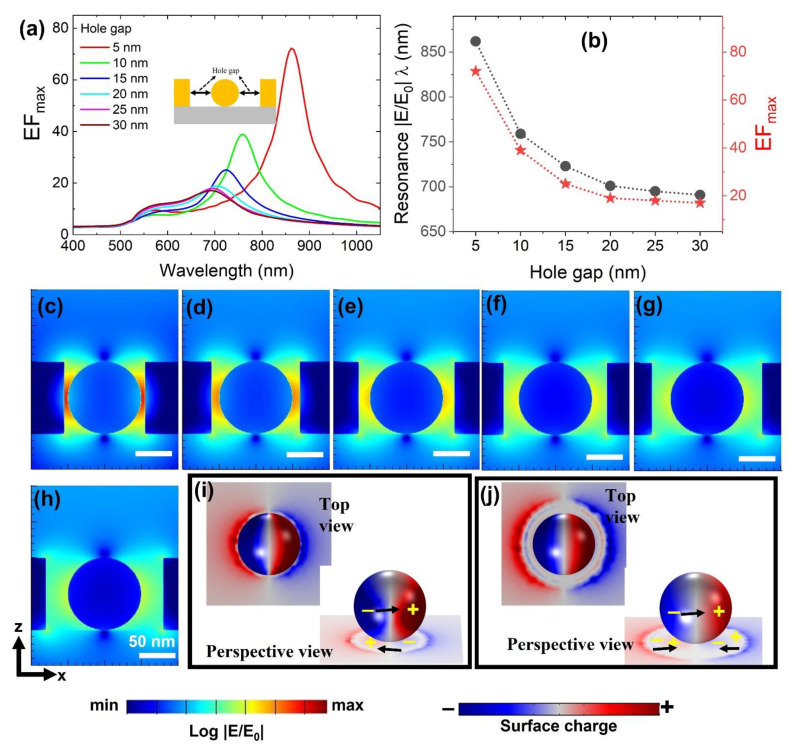
(**a**) Hole-gap size-dependent broadband near-field enhancement EF_max_ spectra with the NP positioned at the bottom of a hole structure and (**b**) corresponding gap mode’s EF_max_ and resonance wavelength positions. (**c**–**h**) Cross-sectional XZ electric field profiles extracted from wavelength positions as seen in Figure 4b as a function of hole-gap size ranging from 5 nm to 30 nm in 5 nm steps. Scale bars equal 50 nm. 3DSCDM profiles taken at hole-gap size of 5 nm (**i**) and 25 nm (**j**). For a perspective view, we hid the hole for clear visibility of charge interactions.

We also considered a possible situation where hole width could be larger than NP diameter (h > d) based upon fabrication errors. To analyze the integrated nanostructure’s geometrical tolerance, a geometrical parameter “hole gap” (Figure 6) was introduced and its influence towards gap-mode based near-field enhancement was evaluated. For this study, a NP positioned at the bottom will be a practical possibility. The hole gap is varied from 5 nm to 30 nm in 5 nm steps, and its influence over broadband near field spectra is shown in Figure 5a. Extracted EF_max_ values from its respective resonance wavelength positions as a function of “hole gap” sizes are plotted in Figure 6b. A decrease in near-field strength is seen when the “hole gap” size increases. The resonance wavelength position of gap mode blue-shifted from 862 nm to 691 nm (171 nm span) with a decrease in EF_max_ from 72 to 17 when the hole-gap size varied from 5 nm to 30 nm. The corresponding cross-sectional XZ electric field profiles taken at their resonance wavelength positions were displayed in Figure 6c–h (5 nm to 30 nm in order). Gap mode properties are observed till the hole-gap size of ~20 nm. Once the plasmonic gap mode became negligibly small or severely deteriorated, EF_max_ resonance wavelength shift and intensity became ~ constant (hole-gap size > 20 nm). To distinguish these characteristics clearly, we extracted 3DSCDM profiles for hole-gap sizes of 5 nm (Figure 6i) and 25 nm (Figure 6i). Even though the surface charge interactions look identical on the NP surface in both cases, a difference is clearly seen on the substrate surface. A dipole mode-like interaction is observed when the hole gap size is 5 nm (Figure 6i). For a hole-gap size of 25 nm, a quadrupole mode appeared (Figure 6j). This explains the why at a 5 nm hole-gap size we can extract better EF_max_, as compared to a 25 nm hole-gap size. A geometrical tolerance of ~20 nm is significantly vital with recent advances in fabrication methods.

## 4. Discussion

In general, NP positioned at the top (the best structure from this work) can be easily assembled upon the success of the hole nanostructure’s identical width (w = d) fabrication. Lithography (nanoparticle lithography or electron-beam lithography) or self-assembly-based methods can facilitate the metallic hole fabrication [45,46,47,48]. After the successful formation of holes, plasmonic NPs can be spin coated. Depending upon hole and NP geometry, top or bottom NP positions can be realized. Enhanced Purcell factor, better EF_max_, and facile fabrication can be realized from the integrated nanostructure. Even though free movements of NP inside the hole can be useful in interesting sensing and trapping studies, NP positioned on the top of the hole will yield better near-field enhancement properties supported by brighter gap modes [49,50,51]. The benefits of our integrated nanostructure can be applied to various applications including catalytic devices. A strong coupling between the plasmon and optical modes will be attractive in facilitating catalytic reactions. As seen from our best-integrated nanostructure (top NP position), superior EF_max_ are extracted as enhanced plasmonic (brighter) mode interactions between the NP and hole occured. Furthermore, enhancement in quantum efficiency will be possible on the basis of strong coupling of plasmonic mode(s) over that of decoupled conditions [52,53,54,55,56,57]. Precisely designed plasmonic structures are required to effectively promote catalytic reactions. The plasmonic structure concentrates the absorbed energy on far-field or near-field enhancement depending on the morphological characteristics of the structure. When we achieve dominant near-field enhancement properties, the energy absorbed by the plasmonic structure contributes to the catalytic reaction of its surroundings rather than emitting it to the outside. The root cause of this difference is based on the plasmonic mode. However, calculating and interpreting three-dimensional plasmonic modes is not yet widespread. Precise design examples (as reported in this work) help catalytic researchers to make the right choice(s) for using plasmonic structures. In addition to the above benefits, a good geometrical tolerance with a presence of gap-mode characteristics facilitates flexible fabrication. Considering these merits, it is possible to realize an efficient plasmonic nanostructure in an integrated form similar to that of a disk- or cube-based NPOM design [8,30]. Our optical design strategy based on an integrated nanostructure can open various attractive applications supported by a simple fabrication.

## 5. Conclusions

In summary, we had reported a three-dimensional finite-domain time-difference simulation study on a NP–hole on a SiO_2_ substrate (integrated nanostructure) and discussed its plasmonic properties. NP positioned at the top of the hole showed better plasmonic characteristics with an enhanced gap mode capability (a maximum of a ~22-times increase in near field strength) and Purcell factor resolving the individual nanostructure’s disadvantage. Effective geometrical tolerance with the availability of gap mode properties till a hole-gap size of ~20 nm can be realized, facilitating flexible fabrication. In addition to all the above advantages, the probability of positioning an NP on top of a hole can be more straightforward, resulting in a simple fabrication. An efficient plasmonic nanostructure or devices based on this integrated (NP–hole) nanostructure can foster a variety of applications in the field of photocatalysis, energy, sensors, surface-enhanced spectroscopy, and ultrafast optoelectronic applications.

## Data Availability

Not applicable.

## References

[1] Devaraj V., Lee J.-M., Kim Y.-J., Jeong H., Oh J.-W. (2021). Engineering Efficient Self-Assembled Plasmonic Nanostructures by Configuring Metallic Nanoparticle’s Morphology. Int. J. Mol. Sci..

[2] Centurion M., Porter M.A., Kevrekidis P.G., Psaltis D. (2006). Nonlinearity Management in Optics: Experiment, Theory, and Simulation. Phys. Rev. Lett..

[3] Amirjani A., Sadrnezhaad S.K. (2021). Computational Electromagnetics in Plasmonic Nanostructures. J. Mater. Chem. C.

[4] Baumberg J.J., Aizpurua J., Mikkelsen M.H., Smith D.R. (2019). Extreme Nanophotonics from Ultrathin Metallic Gaps. Nat. Mater..

[5] Lee J.-M., Devaraj V., Jeong N.-N., Lee Y., Kim Y.-J., Kim T., Yi S.H., Kim W.-G., Choi E.J., Kim H.-M. (2022). Neural Mechanism Mimetic Selective Electronic Nose Based on Programmed M13 Bacteriophage. Biosens. Bioelectron..

[6] Laible F., Horneber A., Fleischer M. (2021). Mechanically Tunable Nanogap Antennas: Single-Structure Effects and Multi-Structure Applications. Adv. Opt. Mater..

[7] Sun A.Y., Lee Y.-C., Chang S.-W., Chen S.-L., Wang H.-C., Wan D., Chen H.-L. (2021). Diverse Substrate-Mediated Local Electric Field Enhancement of Metal Nanoparticles for Nanogap-Enhanced Raman Scattering. Anal. Chem..

[8] Devaraj V., Lee J.-M., Lee D., Oh J.-W. (2020). Defining the Plasmonic Cavity Performance Based on Mode Transitions to Realize Highly Efficient Device Design. Mater. Adv..

[9] Devaraj V., Baek J., Jang Y., Jeong H., Lee D. (2016). Design for an Efficient Single Photon Source Based on a Single Quantum Dot Embedded in a Parabolic Solid Immersion Lens. Opt. Express.

[10] Jiang N., Zhuo X., Wang J. (2018). Active Plasmonics: Principles, Structures, and Applications. Chem. Rev..

[11] Guimbao J., Weituschat L.M., Montolio J.M.L., Postigo P.A. (2021). Enhancement of the Indistinguishability of Single Photon Emitters Coupled to Photonic Waveguides. Opt. Express.

[12] Gellé A., Jin T., de la Garza L., Price G.D., Besteiro L.V., Moores A. (2020). Applications of Plasmon-Enhanced Nanocatalysis to Organic Transformations. Chem. Rev..

[13] Kim S., Jang M.S., Brar V.W., Tolstova Y., Mauser K.W., Atwater H.A. (2016). Electronically Tunable Extraordinary Optical Transmission in Graphene Plasmonic Ribbons Coupled to Subwavelength Metallic Slit Arrays. Nat. Commun..

[14] Lee T., Jang J., Jeong H., Rho J. (2018). Plasmonic- and Dielectric-Based Structural Coloring: From Fundamentals to Practical Applications. Nano Converg..

[15] Kristensen A., Yang J.K.W., Bozhevolnyi S.I., Link S., Nordlander P., Halas N.J., Mortensen N.A. (2016). Plasmonic Colour Generation. Nat. Rev. Mater..

[16] Smith D.R., Pendry J.B., Wiltshire M.C.K. (2004). Metamaterials and Negative Refractive Index. Science.

[17] Lee J.-M., Choi J.W., Jeon I., Zhu Y., Yang T., Chun H., Shin J., Park J., Bang J., Lim K. (2021). High Quantum Efficiency and Stability of Biohybrid Quantum Dots Nanojunctions in Bacteriophage-Constructed Perovskite. Mater. Today Nano.

[18] Kravets V.G., Kabashin A.V., Barnes W.L., Grigorenko A.N. (2018). Plasmonic Surface Lattice Resonances: A Review of Properties and Applications. Chem. Rev..

[19] Mejía-Salazar J.R., Oliveira O.N. (2018). Plasmonic Biosensing. Chem. Rev..

[20] Abouelela M.M., Kawamura G., Matsuda A. (2021). A Review on Plasmonic Nanoparticle-Semiconductor Photocatalysts for Water Splitting. J. Clean. Prod..

[21] Devaraj V., Lee J.-M., Oh J.-W. (2020). Influence of Cavity Geometry towards Plasmonic Gap Tolerance and Respective Near-Field in Nanoparticle-on-Mirror. Curr. Appl. Phys..

[22] Ringe E. (2020). Shapes, Plasmonic Properties, and Reactivity of Magnesium Nanoparticles. J. Phys. Chem. C.

[23] Mann M.E., Yadav P., Kim S. (2021). Colloidal Plasmonic Nanocubes as Capacitor Building Blocks for Multidimensional Optical Metamaterials: A Review. ACS Appl. Nano Mater..

[24] Fan X., Zheng W., Singh D.J. (2014). Light Scattering and Surface Plasmons on Small Spherical Particles. Light Sci. Appl..

[25] Chu S., Chu S., Liang Y., Liang Y., Yuan H., Gao H., Yu L., Wang Q., Peng W., Peng W. (2020). Plasmonic Hybridization Generation in Self-Aligned Disk/Hole Nanocavities for Multi-Resonance Sensing. Opt. Express.

[26] Xiang H., Wang Z., Xu L., Zhang X., Lu G. (2020). Quantum Plasmonics in Nanorods: A Time-Dependent Orbital-Free Density Functional Theory Study with Thousands of Atoms. J. Phys. Chem. C.

[27] Devaraj V., Lee J.-M., Oh J.-W. (2018). Distinguishable Plasmonic Nanoparticle and Gap Mode Properties in a Silver Nanoparticle on a Gold Film System Using Three-Dimensional FDTD Simulations. Nanomaterials.

[28] Devaraj V., Jeong N.-N., Lee J.-M., Hwang Y.-H., Sohn J.-R., Oh J.-W. (2019). Revealing Plasmonic Property Similarities and Differences between a Nanoparticle on a Metallic Mirror and Free Space Dimer Nanoparticle. J. Korean Phys. Soc..

[29] Devaraj V., Lee J.-M., Adhikari S., Kim M., Lee D., Oh J.-W. (2020). A Single Bottom Facet Outperforms Random Multifacets in a Nanoparticle-on-Metallic-Mirror System. Nanoscale.

[30] Devaraj V., Choi J., Kim C.-S., Oh J.-W., Hwang Y.-H. (2018). Numerical Analysis of Nanogap Effects in Metallic Nano-Disk and Nano-Sphere Dimers: High Near-Field Enhancement with Large Gap Sizes. J. Korean Phys. Soc..

[31] Johnson P.B., Christy R.W. (1972). Optical Constants of the Noble Metals. Phys. Rev. B.

[32] Brickdale C., Buchanan A., Butterworth A.R., Chalmers M.D., Clay W.G., Craies W.F., Shepheard W., Palik E.D. (1997). List of Contributors for Volume II. Handbook of Optical Constants of Solids.

[33] Movsesyan A., Muravitskaya A., Castilla M., Kostcheev S., Proust J., Plain J., Baudrion A.-L., Vincent R., Adam P.-M. (2021). Hybridization and Dehybridization of Plasmonic Modes. J. Phys. Chem. C.

[34] Kongsuwan N., Demetriadou A., Horton M., Chikkaraddy R., Baumberg J.J., Hess O. (2020). Plasmonic Nanocavity Modes: From Near-Field to Far-Field Radiation. ACS Photonics.

[35] Juodėnas M., Peckus D., Tamulevičius T., Yamauchi Y., Tamulevičius S., Henzie J. (2020). Effect of Ag Nanocube Optomechanical Modes on Plasmonic Surface Lattice Resonances. ACS Photonics.

[36] Zhang Y., He S., Guo W., Hu Y., Huang J., Mulcahy J.R., Wei W.D. (2018). Surface-Plasmon-Driven Hot Electron Photochemistry. Chem. Rev..

[37] Kasani S., Curtin K., Wu N. (2019). A Review of 2D and 3D Plasmonic Nanostructure Array Patterns: Fabrication, Light Management and Sensing Applications. Nanophotonics.

[38] Sheena T.S., Devaraj V., Lee J.-M., Balaji P., Gnanasekar P., Oh J.-W., Akbarsha M.A., Jeganathan K. (2020). Sensitive and Label-Free Shell Isolated Ag NPs@Si Architecture Based SERS Active Substrate: FDTD Analysis and in-Situ Cellular DNA Detection. Appl. Surf. Sci..

[39] Kleinman S.L., Sharma B., Blaber M.G., Henry A.-I., Valley N., Freeman R.G., Natan M.J., Schatz G.C., Van Duyne R.P. (2013). Structure Enhancement Factor Relationships in Single Gold Nanoantennas by Surface-Enhanced Raman Excitation Spectroscopy. J. Am. Chem. Soc..

[40] Huang Y., Ringe E., Hou M., Ma L., Zhang Z. (2015). Near-Field Mapping of Three-Dimensional Surface Charge Poles for Hybridized Plasmon Modes. AIP Adv..

[41] David C., García de Abajo F.J. (2014). Surface Plasmon Dependence on the Electron Density Profile at Metal Surfaces. ACS Nano.

[42] Krasnok A.E., Slobozhanyuk A.P., Simovski C.R., Tretyakov S.A., Poddubny A.N., Miroshnichenko A.E., Kivshar Y.S., Belov P.A. (2015). An Antenna Model for the Purcell Effect. Sci. Rep..

[43] Huh J.-H., Lee J., Lee S. (2018). Comparative Study of Plasmonic Resonances between the Roundest and Randomly Faceted Au Nanoparticles-on-Mirror Cavities. ACS Photonics.

[44] Langer J., Jimenez de Aberasturi D., Aizpurua J., Alvarez-Puebla R.A., Auguié B., Baumberg J.J., Bazan G.C., Bell S.E.J., Boisen A., Brolo A.G. (2020). Present and Future of Surface-Enhanced Raman Scattering. ACS Nano.

[45] Wen T., Booth R.A., Majetich S.A. (2012). Ten-Nanometer Dense Hole Arrays Generated by Nanoparticle Lithography. Nano Lett..

[46] Najiminaini M., Vasefi F., Kaminska B., Carson J.J.L. (2011). Optical Resonance Transmission Properties of Nano-Hole Arrays in a Gold Film: Effect of Adhesion Layer. Opt. Express.

[47] Han J., Devaraj V., Kim C., Kim W.-G., Han D.-W., Hong S.W., Kang Y.-C., Oh J.-W. (2018). Fabrication of Self-Assembled Nanoporous Structures from a Self-Templating M13 Bacteriophage. ACS Appl. Nano Mater..

[48] Ung T.P.L., Jazi R., Laverdant J., Fulcrand R., des Francs G.C., Hermier J.-P., Quélin X., Buil S. (2020). Scanning the Plasmonic Properties of a Nanohole Array with a Single Nanocrystal Near-Field Probe. Nanophotonics.

[49] Cecchini M.P., Wiener A., Turek V.A., Chon H., Lee S., Ivanov A.P., McComb D.W., Choo J., Albrecht T., Maier S.A. (2013). Rapid Ultrasensitive Single Particle Surface-Enhanced Raman Spectroscopy Using Metallic Nanopores. Nano Lett..

[50] Kerman S., Chen C., Li Y., Roy W.V., Lagae L., Dorpe P.V. (2015). Raman Fingerprinting of Single Dielectric Nanoparticles in Plasmonic Nanopores. Nanoscale.

[51] Maccaferri N., Vavassori P., Garoli D. (2021). Magnetic Control of Particle Trapping in a Hybrid Plasmonic Nanopore. Appl. Phys. Lett..

[52] Mubeen S., Lee J., Singh N., Krämer S., Stucky G.D., Moskovits M. (2013). An Autonomous Photosynthetic Device in Which All Charge Carriers Derive from Surface Plasmons. Nat. Nanotechnol..

[53] Mascaretti L., Dutta A., Kment Š., Shalaev V.M., Boltasseva A., Zbořil R., Naldoni A. (2019). Plasmon-Enhanced Photoelectrochemical Water Splitting for Efficient Renewable Energy Storage. Adv. Mater..

[54] Csete M., Szalai A., Csapó E., Tóth L., Somogyi A., Dékány I. (2014). Collective Plasmonic Resonances on Arrays of Cysteine-Functionalized Silver Nanoparticle Aggregates. J. Phys. Chem. C.

[55] DuChene J.S., Tagliabue G., Welch A.J., Cheng W.-H., Atwater H.A. (2018). Hot Hole Collection and Photoelectrochemical CO_2_ Reduction with Plasmonic Au/p-GaN Photocathodes. Nano Lett..

[56] Shi X., Ueno K., Oshikiri T., Sun Q., Sasaki K., Misawa H. (2018). Enhanced Water Splitting under Modal Strong Coupling Conditions. Nat. Nanotechnol..

[57] Cortés E., Besteiro L.V., Alabastri A., Baldi A., Tagliabue G., Demetriadou A., Narang P. (2020). Challenges in Plasmonic Catalysis. ACS Nano.

